# ^19^F-NMR Probing of Ion-Induced Conformational Changes in Detergent-Solubilized and Nanodisc-Reconstituted NCX_Mj

**DOI:** 10.3390/ijms25136909

**Published:** 2024-06-24

**Authors:** Khiem Nguyen, Tali Strauss, Bosmat Refaeli, Reuben Hiller, Olga Vinogradova, Daniel Khananshvili

**Affiliations:** 1 Department of Pharmaceutical Sciences, School of Pharmacy, University of Connecticut, Storrs, CT 06269, USA; 2 Department of Physiology and Pharmacology, Faculty of Medicine, Tel-Aviv University, Tel Aviv 69978, Israel

**Keywords:** NCX, sodium–calcium exchanger, ^19^F-NMR, ion-induced conformational changes, alternative access mechanism, antiporter, ion transport mechanisms

## Abstract

Consecutive interactions of 3Na^+^ or 1Ca^2+^ with the Na^+^/Ca^2+^ exchanger (NCX) result in an alternative exposure (access) of the cytosolic and extracellular vestibules to opposite sides of the membrane, where ion-induced transitions between the outward-facing (OF) and inward-facing (IF) conformational states drive a transport cycle. Here, we investigate sub-state populations of apo and ion-bound species in the OF and IF states by analyzing detergent-solubilized and nanodisc-reconstituted preparations of NCX_Mj with ^19^F-NMR. The ^19^F probe was covalently attached to the cysteine residues at entry locations of the cytosolic and extracellular vestibules. Multiple sub-states of apo and ion-bound species were observed in nanodisc-reconstituted (but not in detergent-solubilized) NCX_Mj, meaning that the lipid-membrane environment preconditions multiple sub-state populations toward the OF/IF swapping. Most importantly, ion-induced sub-state redistributions occur within each major (OF or IF) state, where sub-state interconversions may precondition the OF/IF swapping. In contrast with large changes in population redistributions, the sum of sub-state populations within each inherent state (OF or IF) remains nearly unchanged upon ion addition. The present findings allow the further elucidation of structure–dynamic modules underlying ion-induced conformational changes that determine a functional asymmetry of ion access/translocation at opposite sides of the membrane and ion transport rates concurring physiological demands.

## 1. Introduction

The cell-membrane Na^+^/Ca^2+^ exchanger proteins (NCXs) mediate an electrogenic ion exchange (3Na^+^:1Ca^2+^) in prokaryotic and eukaryotic cells to handle ion signaling/homeostasis events in health and disease [1,2,3,4]. For example, the cardiac and neuron/glia NCX isoform/splice variants shape dynamic swings in Ca^2+^ to tune spatiotemporal assets of excitation–contraction coupling and neurotransmitter secretion/reuptake [5,6,7,8]. The selective pharmacological targeting of disease-related NCX isoform/splice variants could benefit many biomedical applications. However, this intervention is hampered due to an incomplete understanding of structure-based mechanisms [9,10,11]. The X-ray and cryo-EM structures of prokaryotic and mammalian NCXs [12,13,14,15] provided new opportunities to resolve the structure–dynamic determinants of ion transport and regulation, although the factors causing the functional and regulatory variances among NCX variants (contributing to cell-specific functions) remain unresolved [9,16,17,18].

In contrast with ion channels, the “secondary active transporters” (antiporters, symporters, and uniporters) comply with the alternative access paradigm according to which the ligand (ion)-binding domain undergoes an alternative exposure to opposite sides of the membrane—so, successive transitions between the outward-facing (OF) and inward-facing (IF) states accomplish the ion transport cycle [19,20,21,22]. Although the paradigm of alternative access is generally accepted, the structure–dynamic transitions along the transport cycle might differ in distinct sub-types of secondary active transporters, since the OF/IF swapping may occur either in the absence and/or presence of the ligand [20,23]. For example, in antiporters (like NCXs), the ligand (ion) interactions with respective transport site(s) are obligatory to induce the OF/IF swapping [20,23]. In contrast with antiporters, symporters and uniporters can perform the OF/IF shuttling either in the absence or presence of a ligand/ion [20,21,22,23]. The underlying structure–dynamic mechanisms remain unresolved even for transporters with known structure since available structural snapshots cannot provide a full picture of functionally important intermediates contributing to the transport cycle.

The archaeal NCX_Mj is an excellent prototype for resolving the structure-based mechanisms of ion-induced conformations owned by NCX and similar antiporter systems due to the following reasons [9,10,11,12,13,14,15,16,24]: (a) the ion-coordinating residues (at transport sites) of NCX_Mj are highly conserved among prokaryotic and eukaryotic NCXs; (b) in contrast with mammalian NCXs, NCX_Mj lacks any regulatory domains; and (c) high-resolution crystal structures of NCX_Mj are available. The crystal structure (0.9 Å resolution) of NCX_Mj depicts ten transmembrane helices (TM1–10), where TM1–5 and TM6–10 form two inversely oriented hubs with inverted two-fold symmetry [12,13]. Prokaryotic and mammalian NCXs (in similarity with the Ca^2+^/CA antiporters) contain highly conserved α_1_ (TM2/TM3) and α_2_ (TM7/TM8) repeats that generate an ion-passageway entity with four binding sites: S_ext_, S_mid_, S_int_, and S_Ca_ [12,13,25,26,27,28], thereby suggesting that NCXs might share a common basis for ion-induced alternative access [12,13,25,26,27,28]. Structure-based mutational analysis of ion transport activities in NCX_Mj [29,30,31], in conjunction with MD simulations [32] and the follow-up crystallographic studies [13], have established that in the OF state, NCX_Mj binds either 3Na^+^ (at S_ext_, S_int_, and S_Ca_) or 1Ca^2+^ (at S_Ca_). According to this model, the S_ext_ and S_int_ sites are Na^+^ selective, whereas the S_Ca_ site can be occupied by either Ca^2+^ or Na^+^ (at different stages of the transport cycle). The functional role of the S_mid_ site remains unclear, since according to mutational studies and MD simulations, the S_mid_ site can ligate a water molecule but not Na^+^ or Ca^2+^ [29,30,31,32]. At this end, it remains unclear how the interaction of 3Na^+^ or 1Ca^2+^ with respective sites induces ion-bound sub-states within the major (OF or IF) conformational states. This issue is of fundamental interest since population redistributions of conformational sub-states may precondition the population of OF and IF sub-states directly involved in ion-induced alternative access.

**Figure 1:**
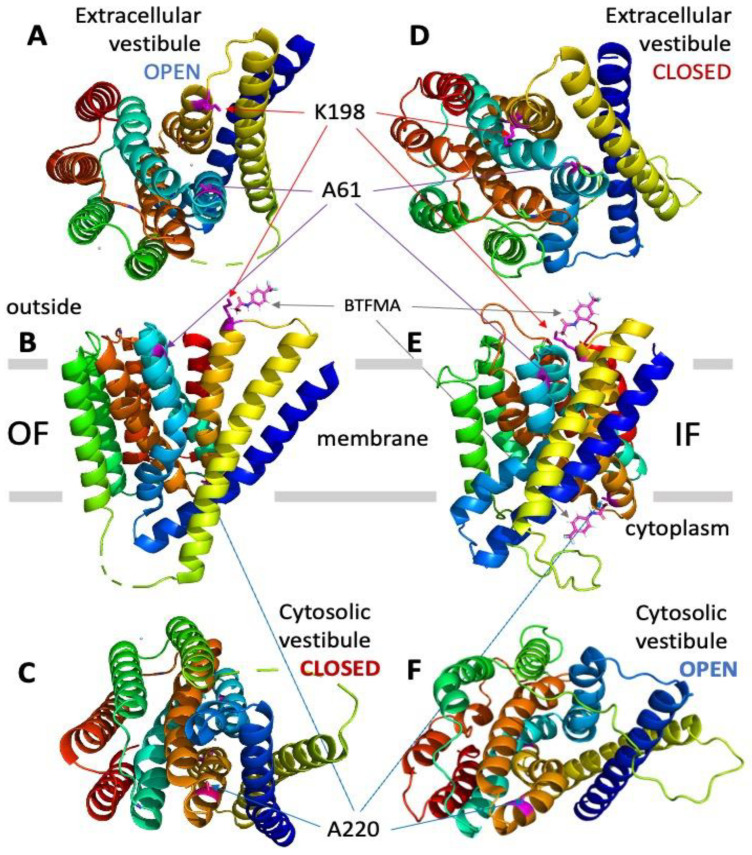
Assignment of target positions for introducing cysteine residues at the cytosolic and extracellular vestibules of NCX_Mj to perform site-directed covalent labeling with the ^19^F probe (BTFMA). The OF (panels **A**–**C**) and IF (panels **D**–**F**) states of NCX_Mj are presented with the positions of target cysteine residues for covalent labeling with BTFMA. The OF structure of NCX_Mj is based on the X-ray data, whereas the IF structure of NCX_Mj was derived using computer-added modeling (for details, see Section 4). Panels **A** and **D** show the NCX_Mj structure from the extracellular viewpoint, while panels **C** and **F** show the NCX_Mj structure from the cytosolic side. The BTFMA moiety is shown near the mutated residues (for labeling) to visualize the size of the ^19^F probe.

The recently discovered cryo-EM structures of the cardiac NCX1.1 [14] and the kidney NCX1.3 [15] revealed that the structural organization of four ion-binding sites and twelve ion-coordinating residues (within the ion-binding pocket) are highly conserved in NCX_Mj, NCX1.1, and NCX1.3. Thus, the prokaryotic and mammalian NCXs might share a common mechanism of ion-induced alternative access (Figure 1A,B), although NCX_Mj (like other prokaryotic NCXs) lacks the regulatory mechanisms owned by eucaryotic NCXs. The X-ray snapshots of NCX_Mj [12,13] in conjunction with HDX-MS [31,33] and functional analyses [29,30,31,32] and MD simulations [32,34], supported the notion that ion-induced “subtle” conformational changes in NCX and similar proteins (belonging to the superfamily of Ca^2+^/CA antiporters) promote the sliding of the two-helix (TM1/TM6) cluster (on the protein surface) toward IF/OF swapping. This common structural module of ion-induced alternating access could be shared by a huge superfamily of Ca^2+^/CA antiporters [12,13,25,26,27,28]. It remains unclear how manifold interconversions between different sub-states in Ca^2+^/CA antiporters can lead to the OF/IF swapping, although these proteins own distinct ion selectivity and stoichiometry of ion binding/transport. How the underlying structure–dynamic mechanisms can contribute to huge variances in the turnover rates and apparent affinities of Na^+^ and Ca^2+^ accessibility to the cytosolic and extracellular vestibules remains unclear [9,10,11,24,29,31].

Previous studies have shown that the detergent-solubilized WT NCX_Mj and its 5L6-8 derivative adopt the OF and IF conformations, respectively; therefore, the HDX-MS analyses of detergent-solubilized WT and 5L6-8 provided indispensable information on Na^+^ and Ca^2+^ interactions with respective sites in the OF or IF conformation [9,10,11,30,31,33]. However, these studies have established that the detergent-solubilized WT or 5L6-8 NCX_Mj cannot perform the OF/IF swapping. Even though HDX-MS analyses of NCX_Mj provided useful information on local conformational changes at ion-binding sites in the OF (WT) and IF (5L6-8) conformations [30,31,33], it remained unclear how the observed conformational states could be related to sub-state conversions that lead to the OF/IF swapping. To overcome this problem, we analyzed nanodisc-reconstituted NCX_Mj using HDX-MS. However, we faced technical obstacles, since lipids dramatically decrease the efficiency of proteolytic digestion of NCX_Mj, thereby making mass-spectra analysis of peptides highly infeasible. In search of an alternative approach, we considered combining ^19^F-NMR techniques [35,36,37,38,39,40] with nanodisc reconstitution techniques of membrane proteins [41,42,43,44,45,46] to analyze ion-induced conformational changes in NCX_Mj. This approach is exciting since lipid compositions can characteristically modulate protein intercalation into nanodiscs [42,45,46]. For example, recent studies have demonstrated that arachidonic acid promotes the binding of 5-lipoxygenase (5LO) to FLAP-nanodiscs independently of Ca^2+^, highlighting the significance of specific lipid interactions in protein complex formation [42].

Potentially, ^19^F NMR could be an indispensable approach for studying ligand-induced conformational changes, because a small-size probe is extremely sensitive to a local environment (water activity) in the lack of endogenous background signals [37,38,39,40,47,48,49,50,51,52]. Notably, a combination of membrane mimetic techniques [43,44,45,46] with ^19^F NMR has the potential to detect ligand-induced conformational changes even in large-size membrane proteins [49,50,51,52,53,54,55,56]. Notably, structure-based MD simulations suggest that “water activity” at the cytosolic and extracellular vestibules of NCX_Mj may significantly fluctuate when adopting the open and close conformations upon ion-induced OF/IF transitions [13,32,34].

To investigate ion-induced conformational changes in NCX_Mj, we applied here the ^19^F-NMR analysis of detergent-solubilized and nanodisc-reconstituted preparations of NCX_Mj. For this purpose, the ^19^F probe (BTFMA) was covalently attached to the thiol group of cysteine residues, the positions of which were selected based on structural information. The site-directed replacements of native residues by cysteine were performed at the entry positions of the extracellular and cytosolic vestibules to detect ion-induced sub-state transitions within two major (OF and IF) states. Only the NCX_Mj cys-mutants, having ion-transport activity, were used for BTFMA labeling and ^19^F-NMR analysis. Multiple conformational sub-states were observed in nanodisc-reconstituted (but not in detergent-solubilized) NCX_Mj within each major (OF or IF) state, revealing that a lipid membrane environment is obligatory for setting an assembly of functionally relevant conformational sub-states, thereby bringing about the ion-induced coupling of alternative access.

## 2. Results

### 2.1. Structure–Functional Prerequisites for Site-Directed Labeling of NCX_Mj cys-Mutants

The structure-based replacements of single residues by cysteine were carefully chosen based on the X-ray structure of NCX_Mj in the OF state [12,13] or computer-added structural modeling of NCX_Mj in the IF state [9,29,30,33] (see also Section 4). As a prerequisite, it was decided that only the cys-mutants retaining the ion exchange activity would be used for covalent ^19^F-probe labeling. For this purpose, the Na^+^/Ca^2+^ exchange activities of cysteine mutants were routinely evaluated by measuring Na^+^-dependent ^45^Ca-uptake in the *E. coli*-derived cell membrane vesicles containing a given (overexpressed) NCX_Mj cys-mutant [29,30,31,32]. This assay system is convenient for assessing ion-flux activities in different mutants, since high protein overexpression levels of WT and NCX_Mj mutants (accounting for 10–15% of the total membrane protein), owning high signal/background ratios of ^45^Ca-uptake detection, allow accurate measurements of K_m_ and V_max_ values for the Na^+^/Ca^2+^ exchange reaction in a given mutant. In the present study, only the cys-mutants retaining the ion-exchange activity (Vmax > 70%, compared with WT NCX_Mj), were used for covalent binding with the ^19^F-probe. Notably, WT NCX_Mj contains two native cysteines (C78 and C80), which are deeply embedded within the protein core of the folded protein. Previous tests have shown that C78 and C80 are inaccessible to externally added sulfhydryl-reactive reagents in the folded protein [31,57]. Consistent with this, no covalent labeling of WT NCX_Mj with the ^19^F probe (BTFMA) was observed in control tests.

To investigate the structure–dynamic features underlying ion-induced conformational changes in the NCX_Mj transporter by ^19^F-NMR, trifluoromethyl phenyl acetamide (BTFMA, ^19^F-containing probe) was covalently attached to the thiols of cysteine residues incorporated into NCX_Mj at predefined positions. These cysteine substitutions have been introduced at the specific points of the extracellular or cytosolic vestibules by site-directed mutagenesis aiming to find out whether the ^19^F-NMR analysis of BTFMA-labeled NCX_Mj can detect and distinguish between the OF vs. IF states in apo and ion-bound states. The purified preparations of NCX_Mj mutants were labeled with 0.2 mM 2-Bromo-N-4-trifluoromethyl phenyl acetamide (BTFMA) by adopting the published protocol [50,51,52] with little modifications (for details, see Section 4).

### 2.2. ^19^F-NMR Cannot Detect Ion-Induced Conformational Changes in Detergent-Solubilized NCX_Mj

The high-resolution crystal structure of NCX_Mj in the OF state [12,13] has been used to choose the single-residue positions for cysteine replacements at entry positions of the cytosolic and extracellular vestibules. For the same purpose, the NCX_Mj structure in the IF state was modeled using the established approaches of computer-aided modeling [29,30,31,32] (see also see Section 4). Initially, two single-point cys-mutants, A61C and A220C, were prepared for BTFMA labeling and ^19^F-NMR analysis, since these two locations are next to the cytosolic (A220C) and extracellular (A61C) entries of the ion-binding pocket and thus may detect ion-induced conformational changes related to the swapping of OF and IF states upon Na^+^ or Ca^2+^ addition. This structure-based labeling of the extracellular and cytosolic vestibules has been applied, since the residue side chains at chosen positions are expected to undergo distinct solvent exposure when adopting the OF vs. IF states, which are anticipated to be detected by ^19^F-NMR (Figure 1). Following this experimental design strategy, we also prepared the double mutant A61C/A220C to detect simultaneous conformational changes at the extracellular and cytosolic vestibules of NCX_Mj.

**Figure 2:**
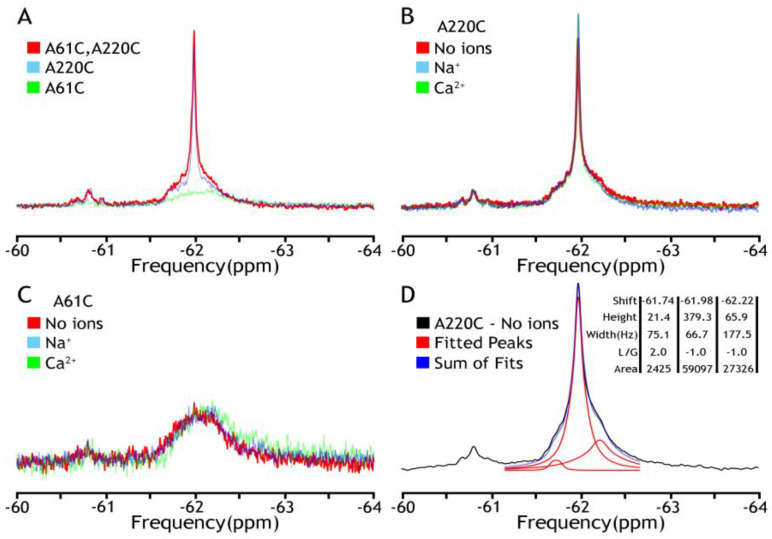
^19^F NMR spectra of NCX_Mj in DDM micelles. The detergent-solubilized (0.25–0.5 mM DDM) preparations of purified A61C, A220C, and A61C/A220C were labeled 0.2 mM BTFMA and then analyzed with ^19^F-NMR. (**A**) The ^19^F signal overlay of three mutants (A61C in green, A220C in blue, A61C/A220C in red) shows the overlap of fluorine labels. Comparison of fluorine signals in the presence (N^a+^ in blue, Ca^2+^ in green) or absence (in red) of ions for A61C (panel **B**) or A220C (panel **C**). (**D**) Deconvolution of A220C (no ions) by MestreNova 14.3 (provided by NMRbox server, spectrum recorded is shown in black, Lorentzian deconvoluted peaks in red, and the sum of fits in blue), which shows the component peaks and their attributes: shift (ppm), height, width (Hz), L/G (peak shape—Lorentzian/Gaussian), and integral area.

At the initial stage of the investigation, the detergent-solubilized (DDM) and nanodisc-reconstituted preparations of BTFMA-labeled NCX_Mj were analyzed by ^19^F-NMR either in the absence or presence of nearly saturating concentrations of Ca^2+^ (1 mM) or Na^+^ (100 mM). The primary goal of these experiments was to compare the ion-induced conformational changes between the detergent-solubilized and nanodisc-reconstituted preparations (as detected by ^19^F-NMR) to evaluate whether the lipid-membrane environment (in nanodisc-reconstituted NCX_Mj) is essential or not for detecting ^19^F-NMR signals in apo and ion-bound states. First, we have noticed a significant difference in the stability of mutants in the presence of detergent. While A220C and A61C/A220C mutants were expressed, purified, and covalently labeled properly, the A61C mutant was unstable with a profound tendency to aggregate. Next, we observed two clusters that overlap ^19^F NMR signals in all the spectra (Figure 2), while representing a major constituent, including a sharp component, around −62 ppm (cluster 1), and a minor broad one around −60.8 ppm (cluster 2). Notably, the A61C mutant showed a shallow broad signal with no sharp component at −62 ppm. Most importantly, in all analyzed mutants, the observed ^19^F-NMR signals were very similar (if not identical) in the presence or absence of Na^+^ or Ca^2+^, thereby revealing that ion interactions with detergent-solubilized NCX_Mj cannot induce OF/IF swapping. These ^19^F-NMR results are highly consistent with previous HDX-MS analyses of detergent-solubilized NCX_Mj, suggesting that Na^+^ or Ca^2+^ interactions with protein can induce a “local” rigidification of the backbone dynamics at respective ion-binding sites without performing the OF/IF transitions [30,31]. Thus, detergent (DDM)-solubilized protein cannot undergo significant conformational changes upon ion binding (in the frame of the alternative access mechanism) as manifested by no changes in population distribution in the absence or the presence of ions (Figure 2B,C). These results could be explained by high TMs flexibility, resembling the molten globule state of helical membrane proteins when lacking a proper membrane-mimicking environment. The spectral deconvolution of A220C peaks from major cluster 1 in the absence of ions is presented in panel D to indicate the measurable heterogeneity of the conformational states even under these “non-optimal” conditions. Based upon these initial findings, a need to make at least two adjustments became eminent: to find a more appropriate representative with the label at the extracellular vestibule and to introduce a better membrane-mimicking environment than a detergent.

### 2.3. ^19^F-NMR Monitors Ion-Induced Conformational Changes in Nanodisc-Reconstituted NCX_Mj

**Figure 3:**
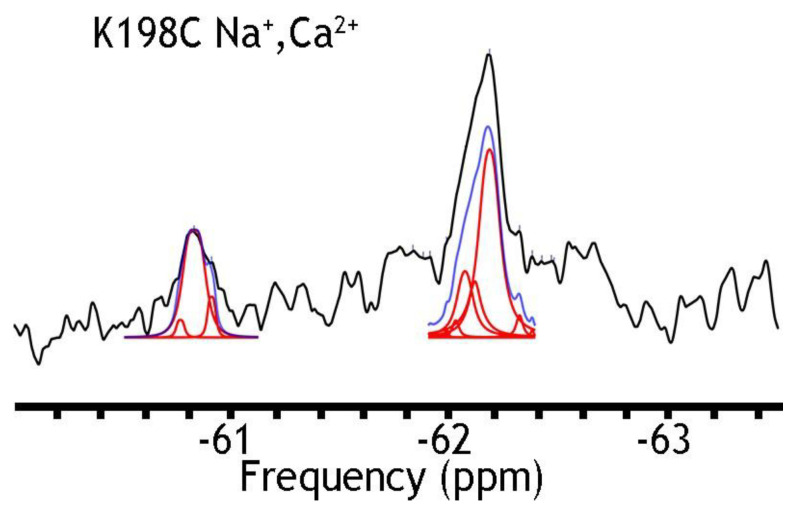
^19^F spectra of nanodisc-reconstituted K198C in the presence of Na^+^ and Ca^2+^ ions. The purified preparation of K198C was labeled with BTFMA and then incorporated into nanodiscs as described in Section 4. The ^19^F-NMR spectra were recorded in the presence of 50 mM Na^+^ and 1 mM Ca^2+^. Spectrum recorded is shown in black, Lorentzian deconvoluted peaks in red, and the sum of fits in blue.

**Figure 4:**
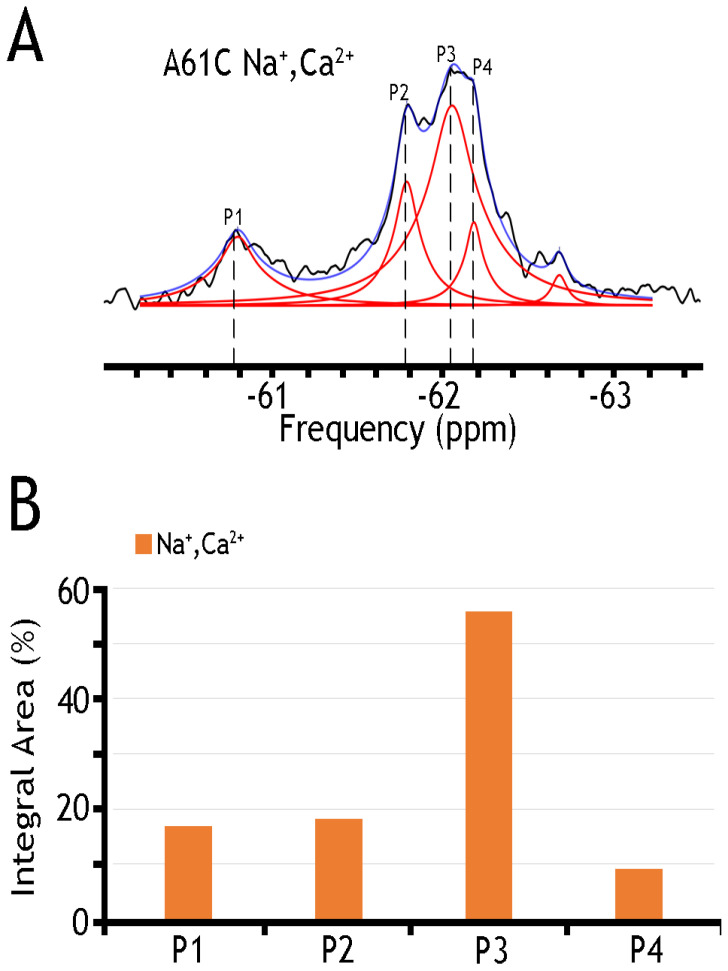
^19^F spectra of nanodisc-reconstituted A61C mutant. The purified preparation of A61C was labeled with BTFMA and then incorporated into nanodiscs (see Section 4). (**A**) ^19^F spectra of BTFMA-labeled A61 mutant (incorporated into nanodiscs) were recorded in the presence of Na^+^ and Ca^2+^ ions. Spectrum recorded is shown in black, Lorentzian deconvoluted peaks in red, and the sum of fits in blue. (**B**) The sub-state populations are quantified by comparing relative integral areas, where the designated sub-states (Ps) are marked on the ^19^F spectrum.

In search of stable mutants, retaining their ion-transport activities, we have checked the K198C mutants (Figure 3). This position has been chosen since the structural [12,13] and functional analysis of single point mutations of NCX_Mj [29,30,31,32,33] have shown that K198 can generate a hydrogen-bonding network, which shifts a steady-state equilibrium in favor of the OF state [1,4,9,29,31]. This might stabilize the OF sub-state populations in the apo or ion-bound form. As in the case of A61C (Figure 4), the K198C mutant appeared unstable, which prevented ^19^F-NMR analysis. However, the K198C/A220C was analyzed by ^19^F-NMR, because this protein retains its structural stability. Since the detergent-solubilized NCX_Mj preparations were not associated with any ion-induced conformational changes (see above), we decided to incorporate the available stable mutants into nanodiscs for further studies to detect any ^19^F-NMR signal that could be associated with ion-induced conformational changes reflecting the OF and IF states under steady-state equilibrium conditions.

**Figure 5:**
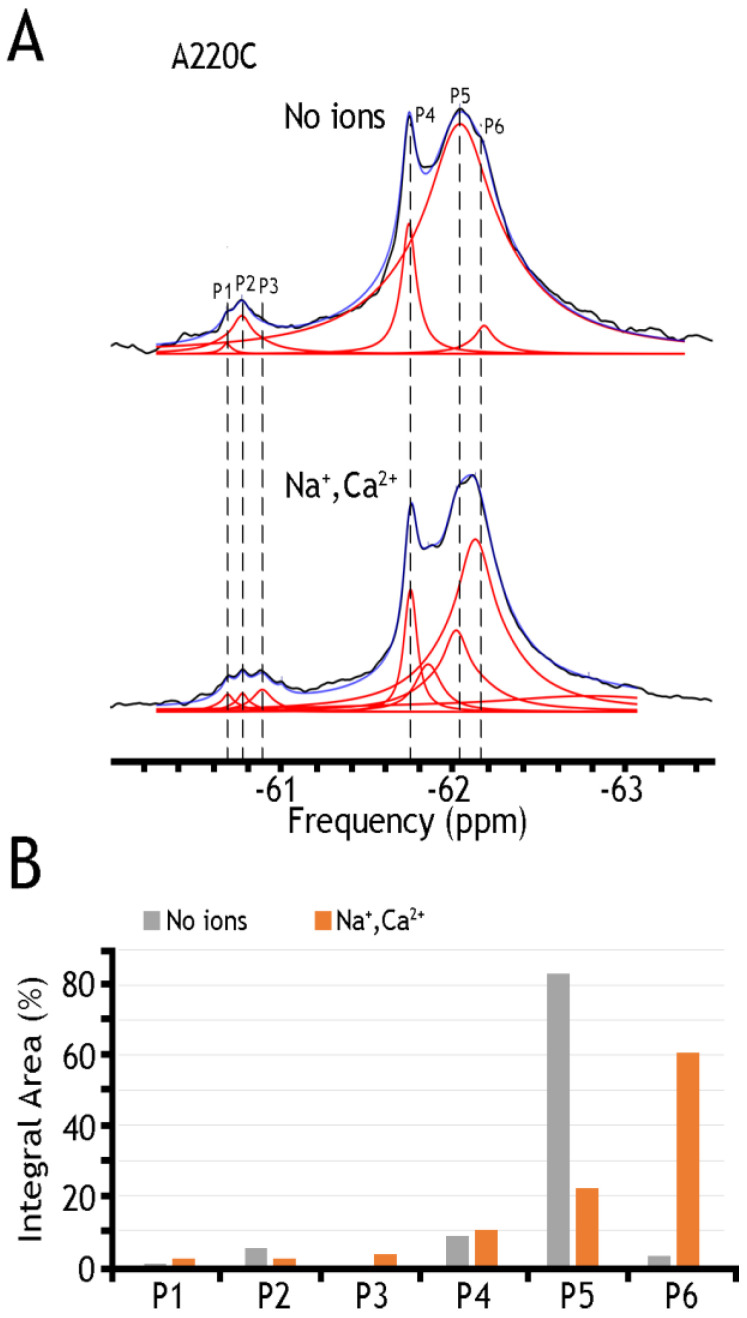
^19^F spectra of nanodisc-reconstituted A220C mutant. The purified preparation of A220C was labeled with BTFMA and then incorporated into nanodiscs to record the ^19^F spectra in the presence or absence of ions. (**A**) ^19^F spectra of A220C mutant incorporated into nanodiscs in the presence or absence of Na^+^ and Ca^2+^ ions. Spectra recorded are shown in black, Lorentzian deconvoluted peaks in red, and the sum of fits in blue. (**B**) Assignment of respective sub-state populations is presented in the presence (N^a+^ and Ca^2+^ in orange) or absence (in gray) of ions. Dashed lines are added for sub-states to show alignment between spectra.

In sharp contrast to detergent-solubilized preparations of BTFMA-labeled NCX_Mj mutants (Figure 2), the nanodisc-reconstituted preparations of the A220C (Figure 5) mutant show significant changes in ^19^F-peaks intensities in the presence of Na^+^ or Ca^2+^, thereby revealing ion-induced changes in populations of different conformations that take place in the presence of a lipid-membrane (but not the detergent) environment. Unfortunately, the observed ^19^F-shifts cannot resolve details of structure–dynamic changes upon ion addition (most probably due to the small dynamic range in chemical shifts of ^19^F-NMR signals and the mobility of the BTFMA-labeled side-chain moiety in the given system). Despite this restriction, the ^19^F-NMR analysis of nanodisc-reconstituted NCX_Mj preparations provides a unique opportunity (for the first time) to detect/analyze multiple conformational states attributing ion-induced swapping of the OF and IF states. These findings demonstrate that the lipid-membrane environment is essential for the OF/IF swapping. The presently applied experimental procedures could be significantly improved by employing more advanced ^19^F-NMR protocols (e.g., using spin probe equipment) and the site-directed labeling of protein with more effective chemical probes (allowing observation of much longer ^19^F-chemical shifts) [47,48] in conjunction with advanced computational approaches, allowing the structure-based calculation (prediction) of ^19^F-chemical shifts in cys-labeled proteins [58,59].

### 2.4. ^19^F-NMR Reveals Manifold Sub-State Transitions within Each of Two Major (OF or IF) State

**Figure 6:**
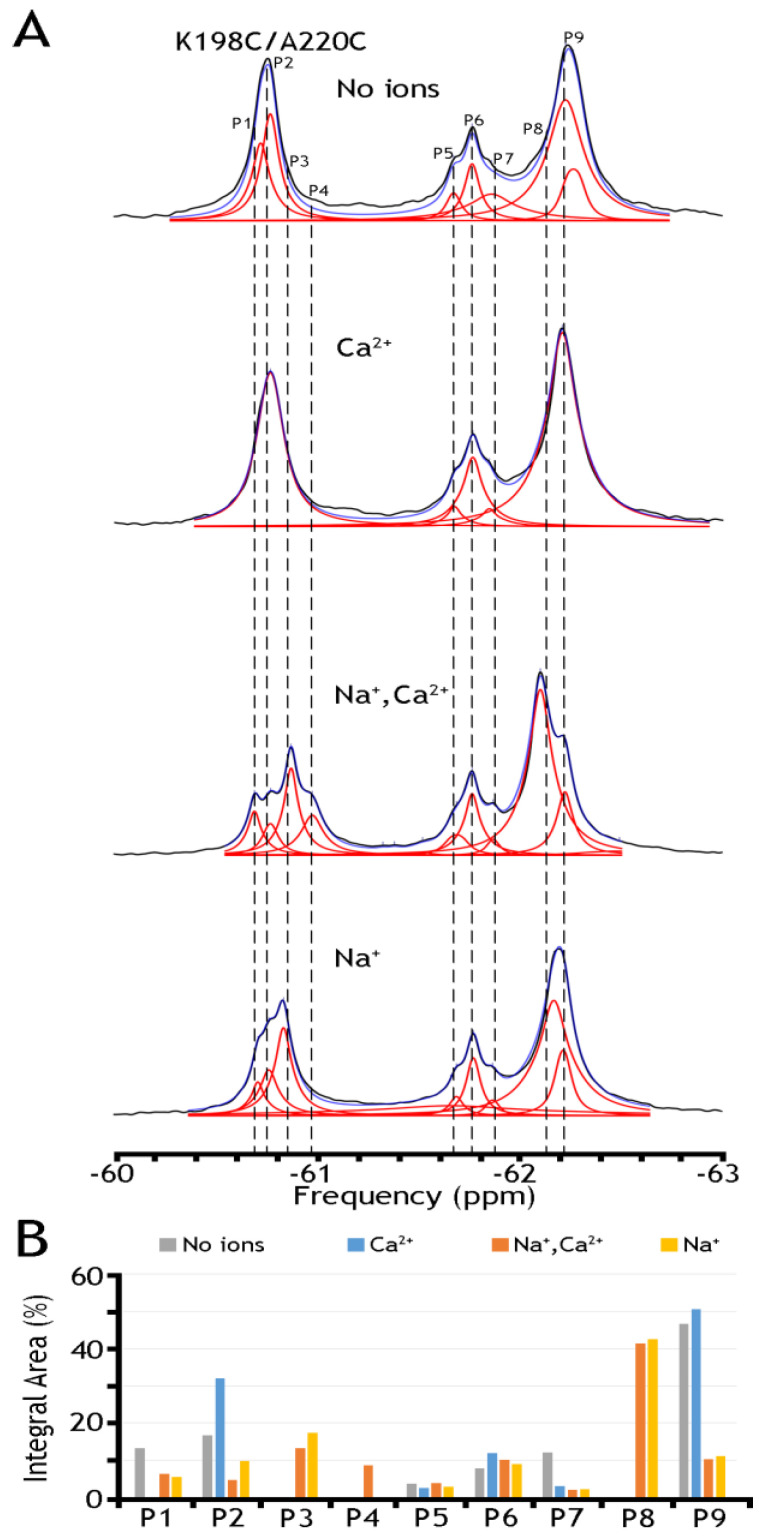
^19^F spectra of nanodisc-reconstituted double mutant K198/A220C. The purified double mutant (K198/A220C) was labeled with BTFMA, and after being reconstituted into nanodiscs, the ^19^F spectra were recorded in the presence or absence of Na^+^ and Ca^2+^. (**A**) ^19^F spectra of nanodisc-reconstituted K198C/A220C in the presence or absence of Na^+^, and/or Ca^2+^ ions. Spectra recorded are shown in black, Lorentzian deconvoluted peaks in red, and the sum of fits in blue. (**B**) Assignment of respective sub-state populations is presented the presence (N^a+^ in yellow, Ca^2+^ in blue, N^a+^ and Ca^2+^ in orange) or absence (in gray) of ions.

A remarkable finding is that nanodisc-reconstituted cys-labeled mutants exhibit characteristic ^19^F-NMR chemical shifts in the absence or presence of Na^+^ or Ca^2+^ (Figure 4, Figure 5 and Figure 6), thereby suggesting that the binding of 3Na^+^ or 1Ca^2+^ with respective sites generates multiple conformational sub-states within each of two major conformational states (OF and IF). The significance of these observations is that the detected assemblies of multi-population sub-states may play a critical role in ion-induced alternative access along the transport cycle in NCX_Mj. The functional relevance of manifold apo and ion-bound intermediates is that the dynamics of their interconversions may predefine the rate-equilibrium relationships of OF/IF transitions, which in turn can limit the turnover rates of the transport cycle and asymmetry of bidirectional ion movements [29,30,31,57]. Thus, the manifold ^19^F-NMR chemical shifts observed here might represent multiple conformational transitions between the apo and ion-bound sub-states when adopting the open, semi-open, and occluded conformations within each major (OF or IF) conformational state. For example, the ^19^F-NMR peaks, observed for A220C (the cytosolic vestibule, Figure 5) and K198C or A61C (the extracellular vestibule, Figure 3 and Figure 4, respectively) look very different, thereby suggesting that water accessibility changes could be quite different at the cytosolic and extracellular vestibules either in the apo or ion-bound states. Notably, the peaks observed for the double K198C/A220C mutant could be considered only as a very approximate superposition of the peaks, since ^19^F signal frequencies of well-defined A220C and more elusive K198C mutants do not match exactly with the ones observed in A220C or K198C. Since the compiled studies [12,13,29,30,31,32,34] revealed that NCX_Mj preferentially adopts the OF conformation either in the apo, Na^+^, or Ca^2+^ bound states, it is reasonable to assume that the cluster 1 (at −62 ppm) represents the most populated assembly of compiling OF sub-states. That leaves cluster 2 (at −60.8 ppm) as the superposition of the peaks reflecting different conformations matching the IF sub-states. More compiled information could be obtained on sub-state interconversions by applying more advanced probes and approaches of ^19^F-NMR [47,48,49,50,51,52,58,59].

To further assess this hypothesis, we have run ^19^F-NMR experiments for the K198C/A220C double mutant, with a better view of the presumably IF state, in the absence of ions or the presence of Na^+^, Ca^2+^, or both ions (Figure 6). In the presence of both ions, we have detected at least five peaks in cluster 1 and as a minimum four peaks in cluster 2. The present observations indicate that P9 (presumably representing an overpopulated OF sub-state without the ion) can be converted into the P8 state upon Na^+^ addition. Similarly, P2, presumably representing the IF sub-state (which is more populated in the apo state), is converted to P3 in the presence of Na^+^. The overall changes in the population of the whole cluster 1 are quite modest again: from ~70.1% (in the absence of ions) to ~68.0% in the presence of Ca^2+^, to ~67.4% in the presence of Na^+^, and to ~67.3% in the presence of both ions. The present observations support the notion that Na^+^-induced changes in the populations of the conformers within either the OF or IF sub-states are more significant than the population changes that we observe between the “global” OF and IF states, therefore supporting the proposed working hypothesis. Even though the present ^19^F-NMR data cannot resolve structure–dynamic transitions underlying the ion-induced conformational changes, the present experimental setup can serve as a good basis for future experimental design and computational modeling, which in turn can provide valuable information on functionally relevant dynamic transitions between distinct sub-states within the OF or IF state. Moreover, the present analysis strongly supports the notion that the site-directed ^19^F probing of NCX_Mj may detect water accessibility changes at opposite entries of the ion-binding pocket, which could be very instrumental for identifying and characterizing functionally relevant sub-state populations limiting the ion transport rates and the intrinsic asymmetry of bidirectional ion binding/transport.

Table 1 presents a numerical presentation of data referring to the populations of A61C, A220C, and K198C/A220C sub-states in the presence of Na^+^ and Ca^2+^ (for a graphical presentation of these data, see Figure 5 and Figure 6, panels B). By taking a closer look into the distribution of A220C peaks, one can notice at least three sharp peaks in cluster 1 and about three broad, less populated peaks in cluster 2. The diversity of the peaks indicates the measurable heterogeneity of the conformational states, meaning that each state, Ca^2+^ or Na^+^ bound, may involve diverse sub-states with variable populations. Upon the addition of Na^+^ and Ca^2+^ ions, the major conversion of P5 into P6 (within the OF sub-states) may occur—whereas correspondingly, the conversion of P2 into P3 may occur (within the IF sub-states) upon ion addition. However, the overall changes in the populations of cluster 1 are insignificant (from ~94.6% to ~92.9%), thereby suggesting that dynamic equilibrium between distinct conformations within the OF or IF state may play an important role in the ion-induced alternating access mechanism.

Our ^19^F-NMR findings underscore the mechanistic significance of multiple sub-state interconversions between the apo and ion-bound states within the OF and IF sub-states—the features that were not previously appreciated. Despite these interconversions within the OF or IF sub-states, the overall OF/IF ratio remains unchanged upon the Na^+^ or Ca^2+^ addition. The mechanism-related significance of these observations deserves further experimental and theoretical elucidation since the ion-induced redistribution of sub-states might increase the probability of the OF/IF swapping either in the inward (IF) or outward (OF) direction. Even though the currently available ^19^F-NMR data cannot resolve structure–dynamic details underlying the ion-induced redistribution of sub-state populations, an application of more advanced ^19^F-NMR techniques in conjunction with structure-based computational modeling can provide indispensable information on sub-state conformer interconversions within two major (OF or IF) conformational states as well as on the OF/IF swapping for NCX [9,10,11,60] and similar proteins [61].

## 3. Discussion

Structural [12,13,14], functional [29,31], and biophysical studies, including HDX-MS [30,33,60], FTIR [57,62], and MD simulations [32,34], revealed structure-based conformational changes upon Ca^2+^ or Na^+^ binding to distinct transport sites of NCX, which highlights crucial structural compliances underlying the ion transport activities. These observations elaborated the “sliding” model of alternative access mechanism for a huge superfamily of Ca^2+^/CA antiporters (NCX, NCKX, NCLX, CCX, and CAX), owing to the diverse ion selectivity and number of ion-binding sites [5,9,10,11,25,26,27,28,61,62,63,64]. According to the proposed model, Na^+^, H^+^, K^+^, Ca^2+^, or Li^+^ binding to a given protein facilitates a movement (sliding) of the two-helix bundle (TM1/TM6) over a distance of ~10 Å [1,5,9,10,11,12,13,29,30,31,32,33,34]. The multi-step transitions might involve numerous conformational changes, although one may ask how a succession of “subtle” conformational changes brings about the OF/IF swapping [65,66,67,68,69,70].

Here, we explored solution-state ^19^F-NMR to monitor Na^+^ or Ca^2+^-driven conformational changes in detergent-solubilized and nanodisc-reconstituted preparations of purified NCX_Mj where a primary goal was to detect apo and ion-bound population sub-states preconditioning the OF/IF swapping. For this purpose, the cysteine residues were introduced by site-directed mutagenesis at predefined positions within the extracellular and cytosolic vestibules, and thiol groups were covalently labeled with the ^19^F probe (BTFMA) (Figure 1). The striking observation is that detergent-solubilized and nanodisc-reconstituted NCX_Mj show very different ^19^F signals. Namely, in sharp difference with detergent-solubilized preparations (Figure 2), the ^19^F-NMR spectra of nanodisc-reconstituted BTFMA-labeled NCX_Mj (Figure 3, Figure 4 and Figure 5) exhibit manifold ^19^F chemical shifts (peaks) with different intensities in the presence of Na^+^ or Ca^2+^. These data demonstrate (for the first time) that a membrane-mimetic lipid environment preconditions ion-induced conformational changes, where multiple population sub-states (within the OF and IF states) are settled to facilitate the OF/IF swapping.

Our ^19^F-NMR data are consistent with previous HDX-MS analysis of detergent-solubilized NCX_Mj [30,33,60], suggesting that micelle-associated NCX_Mj cannot undergo the OF/IF swapping. Thus, the lipid-membrane environment preconditions ion-coupled alternative access in NCX_Mj, which is similar to how some other transporter systems perform [20,21,71,72,73]. Therefore, ^19^F-NMR could be a powerful tool for the future investigation of lipid–protein interactions in NCX proteins that no other techniques could deliver. This is especially encouraging, since negatively charged phospholipids [74,75], anionic amphiphiles [76,77], fatty acids [78], phosphatidyl serine [79], cholesterol [80], long-chain acyl CoA [81] and palmitoylation [82] can modulate mammalian NCX activity through unknown molecular mechanisms. The possible interactions of PIP_2_ [16,77] or arachidonic acid [42] with NCX are of general interest, since this may have physiological and pharmacological significance. Even though the variations in lipid composition do not modulate the NCX_Mj-mediated ion-transport rates [24], our ^19^F-NMR analysis demonstrates that the lipid membrane environment is obligatory for NCX_Mj to perform ion-induced conformational changes that might relate to the alternative access mechanism (Figure 2, Figure 3, Figure 4, Figure 5 and Figure 6). These differences in lipid-dependent modulation can account (at least partially) for large variances in the transport rates owned by prokaryotic and mammalian NCX variants [24,29,30,31]. The ^19^F-NMR approaches used here provide new opportunities for studying the underlying mechanisms of lipid-dependent modulation in NCX and similar proteins.

In sharp contrast with other secondary active transporters, the antiporter system (like NCX) can perform the OF/IF swapping only in the ligand (ion)-bound form(s), whereas the interconversion between the OF and IF states in the apo form (without ligand/ion) is thermodynamically forbidden [20,21,32,34]. The only crystal structure of apo NCX_Mj (in the OF state) is available at pH 4 [13], which makes it difficult to extrapolate this structural information to physiologically relevant conformational states. The structure-based computational analysis of conformational free-energy landscapes has described several Na^+^ and Ca^2+^ bound species (contributing to the NCX_Mj-mediated ion transport cycle) while referring to a “single” conformer for either the OF or IF state [34]. Thus, this analysis concluded that no intermediate sub-states occur within the apo OF or IF state, where a large energetic barrier between these two apo states makes the OF/IF swapping highly improbable. In sharp contrast with the apo state, the energy differences between the OF and IF conformers become small in the Na^+^ or Ca^2+^ bound state, which is compatible with a pose of multiple conformational sub-states within each major (OF or IF) state.

Strikingly enough, our ^19^F-NMR analysis of nanodisc-reconstituted A220C (Figure 5) and K198C/A220C (Figure 6) reveals multiple apo sub-states at the extracellular or cytosolic vestibule. Although the existence of apo sub-state populations within the major OF and IF states has never been appreciated, the dynamic features underlying the interconversion between ion-free populations may have a mechanism-based significance. For example, if interconversions between apo sub-states involve “slow” conformational transitions, this may limit (at least partially) the turnover rates of the overall transport cycle. Even though the resolution of currently available ^19^F-NMR data is not high enough to decipher the structure–dynamic details on apo sub-states, a further application of advanced ^19^F-NMR techniques [47,48,49,53] may provide valuable information on apo populations within the OF and IF states.

Notably, ^19^F-NMR analysis of nanodisc-reconstituted A220C (Figure 5) and K198C/A220C (Figure 6) constructs of NCX_Mj discloses significant differences in the conformer populations when comparing the respective apo and ion-bound species in each construct. For both A220C (Figure 5) and K198C/A220C (Figure 6), the differences (between the apo and ion-bound sub-states) are much more prominent and multiphasic in the case of Na^+^ than in the case of Ca^2+^. These observations are highly consistent with the structural information (obtained by X-ray snapshots of ion-bound species in NCX_Mj) describing the extracellular occlusion of 3Na^+^ or 1Ca^2+^ ions in the OF state [13]. More specifically, a high-affinity (K_d_ ~ 5 mM) binding of 2Na^+^ ions to the S_int_ and S_Ca_ sites is followed by low-affinity (K_d_ > 20 mM) binding of Na^+^ to the S_ext_ site, where the binding of the 3rd Na^+^ ion results in the bending of the TM7A/TM7B segment, so the occlusion of all three Na^+^ ions (at the extracellular side) is achieved [13]. In agreement with these X-ray data, HDX-MS analysis of NCX_Mj has identified local (hotspot) conformational changes at the interface of TM7A and TM7B segments upon Na^+^ (but not Ca^2+^) binding, thereby revealing that the Na^+^-dependent bending of the TM7A/TM7B segment accompanies 3Na^+^ occlusion in the OF state [30,33,60]. Even though these structure–dynamic snapshots represent valuable information on ion binding/occlusion events at distinct stages of the transport cycle, the structure–dynamic features of many other sub-states (that potentially may play an important role in ion-coupled alternative access) remain largely unresolved.

An analytical potential of the applied ^19^F-NMR approach stems from the fact that the tested constructs (A61, A220C, and K198C/A220C) contain two major signal constituents (assigned as Clusters 1 and 2), where each major constituent consists of three to five (at least) overlapping peaks (Figure 4, Figure 5 and Figure 6). The first constituent (Cluster 1) signifies a sharp component, around −62 ppm, whereas the second one (Cluster 2) contains a “minor” broad signal around −60.8 ppm. Since the membrane-bound or detergent-solubilized NCX_Mj preferentially adopts the OF conformation in the apo, Na^+^, or Ca^2+^ bound state [12,13,29,30,33,60], it is reasonable to assume that Cluster 1 (at −62 ppm) stands for the most populated assembly of compiling OF sub-states. In contrast, Cluster 2 (at −60.8 ppm) represents a superposition of IF sub-states. For example, A220C depicts at least three sharp peaks in Cluster 1 and about three broad (less populated) peaks in Cluster 2 (Figure 5 and Table 1), thereby revealing a measurable heterogeneity of conformational sub-states in apo, Ca^2+^, and Na^+^ bound species exhibiting variable population profiles. The observed data indicate that the Na^+^ or Ca^2+^ addition results in the major conversion of P5 into P6 (within the OF sub-states), whereas P2 conversion into P3 refers to ion-induced transitions within the IF sub-states. Despite the detectable ion-induced transitions, it is worthwhile to note that the overall changes in the populations of Cluster 1 are insignificant, meaning that dynamic equilibrium within the OF or IF sub-states may predefine a population of ion-bound OF and IF sub-states directly involved in the OF/IF swapping.

To further assess the underlying mechanisms of ion-induced conformational transitions, we analyzed nanodisc-reconstituted K198C/A220C, because this double mutant provides a better overlook of less populated IF sub-states either in the apo or ion-bound states (Figure 6). In the presence of both ions, we have detected at least five peaks in Cluster 1 and four peaks (at least) in Cluster 2. These tests revealed that P9 (presumably representing an overpopulated OF sub-state in the apo state) can be converted into P8 upon Na^+^ addition. Similarly, P2 (presumably representing a more populated apo IF sub-state) can be converted to P3 in the Na^+^-bound form. However, the population redistribution profiles of respective sub-state conformers are somewhat different in the presence of Ca^2+^ (Figure 6). As in the case of A220C (Figure 5), we found that in K198C/A220C, the overall changes in Cluster 1 are quite modest either in the presence of Ca^2+^ or Na^+^ or both ions (Figure 6). Thus, the compiled data reveal significant ion-induced transitions within the OF or IF sub-states, where the overall OF/IF ratio remains enduring under the steady-state conditions of the transport cycle.

The major limitation of the present ^19^F-NMR analysis of the nanodisc-reconstituted NCX_Mj is that it cannot resolve structure–dynamic details underlying the sub-state population transitions with two major (OF and IF) conformational states. The major reason for this technical short is a limited chemical dispersion (implemented in small ^19^F chemical shifts) and the high mobility of the ^19^F-probe (BTFMA) moiety. Even though these shortcomings represent a principal barrier to studying multi-state populations under dynamic equilibria [47,48,49,53], the newly developed monofluoroethyl ^19^F probes can dramatically increase the chemical shift dispersion, thereby providing improved conformational sensitivity and line shape, enabling the detection of previously unresolved states in one-dimensional (1D) ^19^F-NMR spectra. More specifically, the chemical shift dispersion of currently available ^19^F probes (including BDFMA) generally does not exceed 2 ppm, which is compatible with severe resonance overlaps [53], as observed in the present study. In the great improvement of currently available ^19^F-probes, the newly developed cysteine-conjugated monofluoroethyl probe possesses narrow linewidths and ultrahigh sensitivity to ligand-induced conformational changes in proteins with chemical shift dispersion reaching 9 ppm [47,49]. The future application of new monofluoroethyl probes for ^19^F-NMR analysis of nanodisc-reconstituted NCX_Mj cys-mutants may dramatically increase the resolution of chemical shifts observed in the present study. Combining the experimental approaches described in the present work with the emerging ^19^F-NMR approaches [47,48,49,53] and especially suited structure-based computational calculations [58,59] may provide game-changing information on multiple interconversions between sub-state populations in the apo and ion-bound states from the perspective of ion-coupled alternative access.

Another way to improve the structure–dynamic resolution of ^19^F-NMR data (obtained in the present study) is to adopt new experimental approaches of ^19^F-NMR (recently developed for a glutamate transporter homolog) [21,22,47,48] for the future analysis of nanodisc-reconstituted NCX_Mj. This new approach would involve the site-directed cys-^19^F-probe introduction at specific locations of the cytosolic and extracellular vestibules (as carried out in the present work), where a two-his-tag will be attached to a neighboring transmembrane helix (TM3 or TM8) to chelate Ni^2+^ as a paramagnetic probe. This experimental setup allows the Ni^2+^-enhanced detection of ^19^F longitudinal relaxation rates, which can be used for quantifying the ligand (Na^+^ or Ca^2+^)-induced distance changes of the ^19^F probe related to the ion-induced conformational sub-states within and between the OF and IF states. Thus, the presently used experimental approaches provide a good basis for applying more advanced experimental techniques [47,48,49,53].

Even though several NCX blockers (KB-R7943, SEA0400, SN-6, and YM-244769) are currently available [82,83], it is quite clear that besides NCX, these compounds also can interact with other membrane proteins [84,85,86]. Another fundamental obstacle is that the selective pharmacological targeting of tissue-specific NCX variants remains unavailable due to an incomplete understanding of the underlying molecular and cellular mechanisms [1,5,7,9,10,11,60]. Strikingly, the recently disclosed Cryo-EM structure of the kidney NCX1.3 revealed that the SEA0400 binding to the TM2AB segment may stiffen (rigidify) a local structural entity, although according to the original model of the sliding mechanism, the TM2 helix should not undergo significant conformational changes upon ion binding [11,12,13,14,15,25,26,27,32]. To this end, it remains unclear how SEA0400 affects the redistributions of sub-state population assemblies. Thus, the ^19^F-NMR approaches, applied here, could be instrumental in elucidating how SEA0400 (or any other inhibitor) affects sub-state populations of conformer distributions and how this could affect the ion transport rates. In conjunction with structural information, ^19^F-NMR analysis of inhibitor-induced conformational changes may provide useful information for the selective pharmacological targeting of tissue-specific NCX variants, which has huge biomedical significance.

## 4. Materials and Methods

### 4.1. Cloning and Overexpression of WT and Mutant NCX_Mj

DNA encoding the wild-type NCX_Mj was amplified by PCR from a *Methanocaldococcus jannaschii* cDNA library (DSMZ), which was ligated between the NcoI and BamHI restriction sites of a pET-28a plasmid, and the DNA sequence encoding a Tobacco etch virus (TEV) proteolysis site (followed by a 6xHis tag) was appended downstream of the NCX-Mj gene [29,30,31,32,33]. Single-point mutations were introduced by QuickChange mutagenesis (Stratagene, La Jolla, CA, USA) and confirmed by sequencing as previously established [29,32,33]. Expression vectors were transformed into *E. coli* BL21 (DE3) pLysS competent cells, and cells were grown in 2xYT media with antibiotics. When cell cultures reached OD_600_ = 0.5–0.6 at 16 °C, expression was induced by adding IPTG, and cells were harvested 12–16 h after induction. Centrifuged cells were resuspended in storage buffer (50 mM Mops-Tris pH 7.4, 250 mM sucrose, 1 mM EDTA, 1 mM DTT, 100 Units/mL DNase, 1 mM PMSF, 1 mM benzamidine and protease inhibitor cocktail [29,31,32,33] and kept frozen at −80 °C until usage. The typical overexpression levels of WT- and cys-NCX_Mj proteins (8–12% of total membrane proteins) were comparable for the mutants tested here.

### 4.2. Preparation of E. coli Membranes Containing Overexpressed WT or cys-NCX_Mj Proteins

First, 300–400 g of *E. coli* cells (containing overexpressed NCX_Mj cys-mutants) were resuspended in a storage buffer (see above), homogenized in a hand homogenizer, and passed through a 50 mL syringe. Then, the cells were disrupted by passing (three times) the cell suspension through the EmulsiFlex-C3 device (Avestin, Inc, Ottawa, ON, Canada) at 15,000-20,000 psi, and then they were centrifuged at 6000× *g* for 5 min. The crude fraction of membranes was obtained by centrifugation of the supernatant at 150,000× *g* for 1 h. The pellet of crude membranes was resuspended in 50 mM Mops-Tris buffer pH 7.4, 1 mM EDTA, 1 mM DTT, and loaded onto a three-step sucrose gradient (2.02, 1.4, and 0.7 M). After centrifugation at 150,000× *g*, 4 °C for 15 h in the Ti-45 rotor, a brownish layer that appeared between the 0.7 and 1.4 M sucrose layers was collected and diluted 3 times by water [29,31,33]. Membrane vesicles (containing overexpressed WT or mutated NCX_Mj protein) were obtained by centrifugation at 150,000× *g* for 1 h, and the pellets of the membrane vesicles were resuspended in 50 mM Mops-Tris pH 7.4 and 0.25 M sucrose. The suspension of membrane vesicles (3–7 mg protein per mL) was homogenized by a hand homogenizer and passed through the 5 mL syringe to crumble aggregated particles. The homogenized membrane vesicles were flash-frozen in liquid nitrogen and stored at −80 °C until ion-flux assays or protein solubilization/purification purposes.

### 4.3. cys-NCX_Mj-Mediated Ion-Transport Assays Using the Isolated Vesicles Derived from E. coli

The WT- or cys-NCX_Mj-mediated Na^+^/Ca^2+^ and Ca^2+^/Ca^2+^ exchange rates were measured by assaying ^45^Ca-uptake using the isolated membrane vesicles (derived from *E. coli*), which contained the overexpressed WT or cys-NCX_Mj protein [29,30,31,32]. Briefly, Na^+^ (160 mM) or Ca^2+^ (0.25 mM)-loaded vesicles were diluted (at 35 °C) in the assay medium containing 20 mM Mops/Tris, pH 6.5, 100 mM KC1, and 0.2 mM ^45^CaCl_2_, and ^45^Ca-uptake was quenched after 10 s by injecting cold EGTA-buffer into the assay medium [29,31,32,33]. The quenched solutions were immediately filtrated on GF/C filters (Tamar Ltd., Jerusalem, Israel), and the filter-bound radioactivity was quantitatively evaluated using scintillation counting. For non-specific ^45^Ca binding, the radioactivity was measured in the *E. coli*-derived vesicles lacking the overexpressed NCX_Mj protein, and the non-specific (background) signals were subtracted from the signals obtained with the vesicles containing the overexpressed WT or cys-NCX_Mj protein. Due to the high overexpression levels of WT or cys-NCX proteins (8–12% of total membrane protein) in the *E. coli* expression system, the signal/background ratios were usually higher than 7. All other details for measuring the NCX-mediated ^45^Ca-uptake in vesicular preparations were previously described.

### 4.4. Purification of WT and cys-Mutated NCX_Mj Proteins

The WT- and cys-NCX_Mj proteins (overexpressed in the *E. coli* system) were extracted from the isolated membrane vesicles with DDM (bioWORLD) and then purified using the established protocols His-tag affinity column and gel-filtration [29,30,31,32,33]. Briefly, the isolated vesicles (see above) derived from the *E. coli* membranes (containing an overexpressed protein of a given cys-mutant) were extracted with 20 mM DDM (50 mM HEPES pH 7.1, 50 mM NaCl, 12 mM KCl, and 10 mM CaCl_2_), supplemented with DNase (10 µg/mL), 1 mM PMSF (Sigma-Aldrich, St. Louis, MO, USA), 1 mM benzamidine (Sigma- Aldrich, St. Louis, MO, USA) and protease inhibitor cocktail Xpert (GenDEPOT, Baker, TX, USA). After 3–4 h of gentle stirring, the supernatant was centrifuged at 20,000× *g* for 15 min, and the clarified supernatant was loaded onto a Ni^2+^-NTA or Co^2+^ (TALON, Takara Bio Inc, San Jose, CA, USA) column with a flow rate of 0.1 mL/min. The column was washed with 4 mM DDM plus 10 mM imidazole based on 50 mM HEPES pH 7.1, 50 mM NaCl, 12 mM KCl, and 10 mM CaCl_2_, and a given cys-NCX_Mj protein was eluted with 1 mM DDM and 300 mM imidazole with the same components of the basic buffer. The fractions containing a protein were collected, desalted (to eliminate imidazole), and concentrated (5–10 mg protein/mL) with an Amicon Ultra-15 device (a 10 kD cut-off filter, Merk KGaA, Darshadt, Germany). The desalted and concentrated protein sample was digested overnight with TEV protease (1:80 ratio) to remove the His-tag. Following a second passage through a Ni^2+^ or Co^2+^ column, the concentrated protein sample (~0.5 mL) was loaded on the gel-filtration column and subjected to size-exclusion chromatography using a Superdex SD200 Increase column, 10/300 GL (Cytiva, Uppsala, Sweden) pre-equilibrated with 50 mM Hepes, 7.2, 100 mM CsCl, 0.2 mM EGTA and 0.5 mM DDM. The eluted fractions, containing a given cys-NCX_Mj protein, were collected and concentrated (4.5–6.5 mg protein/mL). The purified cys-NCX_Mj proteins (>90% purity, as judged by SDS-PAGE) were flash-frozen in liquid nitrogen and stored at −80 °C until covalent labeling (see below).

### 4.5. Covalent Labeling of Purified cys-NCX_Mj Proteins by BTFMA

The covalent labeling of purified preparations of detergent-solubilized cys-NCX_Mj proteins with a 2-bromo-N-4-trifluoromethyl phenyl acetamide (BTFMA) probe was performed using a previously established protocol describing the labeling of micelle-associated purified membrane protein (A_2A_R) with BTFMA [38,39]. Briefly, a freshly prepared stock solution of BTFMA (0.5–1 M) in DMSO was slowly added to 20–50 µM cys-NCX_Mj to reach a final concentration of 200 μM BTFMA in the labeling buffer (50 mM HEPES, 7.2, 100 mM CsCl, 0.2 mM EGTA, and 0.5 mM DDM). The samples were incubated at 4 °C overnight with gentle agitation. Then, a second aliquot of 200 μM BTFMA was added to the labeling buffer at 4 °C, and after 6–10 h, the incubation was terminated by the removal of unreacted BTFMA. To separate the covalently labeled protein from unreacted BTFMA, the samples were loaded onto a P10 (Cytiva) gravity column (Sephadex G-25) pre-equilibrated with 50 mM HEPES 7.2, 100 mM CsCl, 0.2 mM EGTA, and 0.5 mM DDM. The eluted fractions of BTFMA-labeled NCX-Mj preparations were combined and concentrated (5–6 mg protein/mL) using the Amicon Ultra-4 device (a 10 kD cut-off filter), representing the apo (0.2 mM EGTA) form of a given BTFMA-labeled NCX-Mj protein. The stock solutions of 4 M NaCl or 1 M CaCl_2_ were added to the apo form preparation of BTFMA-labeled NCX-Mj to reach the final concentrations of 100 mM Na^+^ or 2 mM Ca^2+^. The apo, Na^+^- and Ca^2+^-containing samples of BTFMA-labeled NCX-Mj proteins were flash-frozen in liquid nitrogen and stored at −80 °C until reconstitution into nanodiscs.

### 4.6. Reconstitution of BTFMA-NCX_Mj Proteins into Nanodiscs

The purified preparations of BTFMA-labeled NCX_Mj proteins (with 0.25–0.5 mM DDM) were reconstituted into nanodiscs with 50 mM Na^+^ and/or 1 mM Ca^2+^ in a buffer containing 20 mM Tris, pH 7.5, 50 mM NaCl, and 0.5 mM EDTA. To form an optimal size of nanodisc, the D7 construct of the membrane scaffold protein (MSP) was utilized, which was overexpressed and purified according to a published protocol [44,45,46]. The lipid, 1,2-dimyristoyl-sn-glycero-3-phosphocholine (DMPC) was purchased in a powder form (Avanti Polar Lipids, Alabaster, AL, USA), and a stock solution of 50 mM DMPC was prepared by dissolving the lipid in the buffer containing 100 mM Na-cholate. The BTFMA-labeled NCX_Mj proteins, D7, and DMPC were mixed with a ratio of 1:2:80 while yielding the final concentrations of 15 mM Na-cholate and 4 mM DMPC. The mixed solution was allowed to gently agitate on an orbital shaker at room temperature for two hours. The nanodisc formation was initiated by adding the Bio-bead (SM-2) Adsorbent Media (BioRad, Hercules, CA, USA) to the solution with a final ratio of 0.15 g Bio-beads per milliliter. The solution was gently agitated using an orbital shaker for an additional four hours at room temperature. After the detergent removal, the solution was filtered through a 0.45 µm cellulose-acetate filter to remove non-specific precipitates and separate the nanodisc-containing solution from the Bio-beads. Protein concentrator tubes (with a 10 kD cut-off filter) were used to concentrate the tested samples and/or as well as to exchange the salt composition in the buffer.

### 4.7. Nuclear Magnetic Resonance

For ^19^F-NMR analysis, the nanodisc-reconstituted BTFMA-NCX_Mj proteins were equilibrated with the 50 mM HEPES, pH 7.1 buffer, and desired salt composition, as indicated in figure legends. The ^19^F NMR analysis of nanodisc-reconstituted BTFMA-NCX_Mj proteins was performed on a Bruker AVANCE 500 MHz spectrometer (Bruker, Malvern, PA, USA) with a BBFO smart probe at 25 °C. The concentration of BTFMA-NCX_Mj proteins was variable (10–100 µM). The NMR sample contained 9% D_2_O and 0.1 mM trifluoroacetic acid (TFA) as the reference compound. The chemical shifts were referenced by setting TFA to −75.6 ppm. The ^19^F NMR analysis of nanodisc-reconstituted BTFMA-NCX_Mj samples was run directly after the nanodisc preparation. The samples (in the Tris buffer) were kept with Na^+^ and Ca^2+^ ions to ensure the retention of the NCX_Mj protein into the nanodiscs. All other nanodisc samples were in 50 mM HEPES pH 7.1 buffer with or without ions as described in the figure legends. The ^19^F NMR spectra were collected overnight by recording 30,000 scans. The data analysis was processed, and peaks were deconvoluted using MestReNova 14.3, which was provided by the following NMRbox server: https://nmrbox.nmrhub.org (accessed on 19 June 2024).

## Figures and Tables

**Table 1:** Integral areas of each deconvoluted peak in A61C, A220C, and K198C/A220C mutants. The integral area of each deconvoluted peak correlates with an approximate population of the sub-state (represented by the peak at that particular frequency).

A61C		Integral Area (%)			
Label	Shift (ppm)	Na^+^, Ca^2+^			
P1	−60.75	16.80			
P2	−61.75	18.18			
P3	−62.05	55.86			
P4	−62.15	9.14			
**A220C**		**Integral Area (%)**			
Label	Shift (ppm)	Na^+^, Ca^2+^		EGTA (no ions)	
P1	−60.7	2.01		0.44	
P2	−60.8	1.91		4.96	
P3	−60.9	3.16		0.00	
P4	−61.75	10.16		8.56	
P5	−62.05	21.99		83.27	
P6	−62.15	60.76		2.80	
**K198C/A220C**		**Integral Area (%)**			
Label	Shift (ppm)	Na^+^, Ca^2+^	EGTA (no ions)	Na^+^	Ca^2+^
P1/p1	−60.67	6.23	13.20	5.52	0.00
P2/p2	−60.75	4.69	16.64	9.71	32.04
P3/p3	−60.85	13.13	0.00	17.37	0.00
P4	−60.95	8.64	0.00	0.00	0.00
P5	−61.65	3.74	3.64	2.86	2.55
P6/p4	−61.75	10.04	7.83	8.91	11.94
P7	−61.85	1.94	12.10	2.15	2.90
P8/p5	−62.15	41.31	0.00	42.47	0.00
P9/p6	−62.20	10.28	46.59	11.01	50.58

## Data Availability

The NMR software used is available freely from the NMRBox server: https://nmrbox.nmrhub.org (accessed on 19 June 2024). All raw NMR spectra collected are available from the corresponding author (OV) upon request. NMR spectra examples are provided in Figure 2, Figure 3, Figure 4, Figure 5 and Figure 6.
